# Conservative interventions for the treatment of pelvic organ prolapse: A systematic review protocol

**DOI:** 10.1097/MD.0000000000018116

**Published:** 2019-11-22

**Authors:** Yuanjie Sun, Huan Chen, Yan Liu, Ruimin Jiao, Jingxue Yuan, Xuecheng Zhang, Zhishun Liu

**Affiliations:** a Guang’anmen Hospital, China Academy of Chinese Medical Sciences; b Key Laboratory of Chinese Internal Medicine of Ministry of Education, Dongzhimen Hospital, Beijing University of Chinese Medicine; c Beijing University of Chinese Medicine, Beijing, China.

**Keywords:** adult women, pelvic floor muscle training, POP, Prolapse

## Abstract

**Background:**

Pelvic organ prolapse (POP) is the downward descent of vaginal walls, affecting the health of 32% to 76% female patients. Conservative interventions are considered as priority before seeking help from surgery. We plan to make the systematic review to assess the effectiveness of conservative intervention for adult women with POP.

**Methods:**

Studies will be searched in PubMed, EMBASE, and the Cochrane Library from inception to July 2017. Primary outcomes are specific POP-related symptom, severity of prolapse, Prolapse-related, and general health-related quality of life and other non-POP-specific symptoms.

**Results:**

The data will be synthesized if possible using MD, SMD or RR. A descriptive analysis will be made if the data cannot be synthesized.

**Discussion:**

The systematic review might provide solid evidence for the treatment of POP by conservative intervention.

## 1 Introduction

Pelvic organ prolapse (POP), defined by anatomical change, is the descent of 1 or more of the anterior vaginal wall, posterior vaginal wall, the uterus (cervix), or the apex of the vagina (vaginal vault or cuff scar after hysterectomy).^[1]^ The most specific symptom of POP is vaginal bulging,^[2,3]^ and patients may also suffer from urinary, bowel, and sexual symptoms^[4]^. However, the relationship between anatomical change and symptoms is poor, especially in milder stages where typical symptoms may not even appear.^[5]^ In clinic, appropriately 6% patients have bulging feelings in vagina^[6]^ while up to 32% to 76% female patients accompany with loss support of vagina or uterus.^[7,8]^

Treatments might not be provided to POP patients until the symptoms become bothersome.^[9]^ Conservative treatments tend to be considered as priority before patients seek further help from surgery, especially for those at mild-degree of POP, wishing for child, of frail body or unsuitable for surgery.^[3]^ In IUGA/ICS report, conservative interventions for POP are defined as non-surgical and non-pharmacological treatments, mainly including lifestyle intervention, devices and physical therapies.^[2]^ Lifestyle interventions mainly refer to weight loss and avoiding heavy lifting or coughing. Most frequently used devices at present are support pessaries and space filling pessaries. Physical therapies mainly include a variety of physical activity, cognitive behavioral therapy, pelvic floor muscle training, bladder training, bowel habit training, coordination training, biofeedback, electrical muscle stimulation, and others.

Pelvic organs are mainly supported by the levator ani muscle complex and connective tissue.^[10]^ Damage to the integrity of muscular, connective and nerve structures could bring threat to the normal support, which might be induced by childbirth, advancing age, and increasing body-mass index, with vaginal childbirth as the most consistent risk factor.^[4]^ Conservative treatments may reduce intro-abdominal pressure, build up muscle strength and prevent organ from downwards.^[11,12]^

Two systematic reviews^[11,13]^ were published in 2011 and 2013 to assess the effects of conservative treatments for POP. Conclusion was drawn that pelvic floor muscle training, as a physical therapy, was supported by some rigorous trial as effective, but evidence remained limited and no good quality evidence supported other conservative treatments. Since several other RCTs in this area have been published ever since, and innovative therapies may spring up, we plan to make this systematic review to reassess the effects of conservative interventions for adult women with POP.

## 2 Methods

The protocol of the systematic review has been registered on PROSPERO with the number of CRD42019136277 and reported under the guideline of Preferred Reporting Items for Systematic Reviews and Meta-Analyses protocols (PRISMA-P).^[14]^

### 2.1 Criteria for including studies in this review

#### 2.1.1 Types of studies

We will only consider randomized controlled trials. Quasi-RCTs, cross-over RCTs and cluster RCTs will all be excluded.

#### 2.1.2 Types of participants

- Female patients at age over 18 years will be included;
- Patients diagnosed as POP will be included, without limit of diagnostic criteria or prolapse segments. They may suffer from anterior vaginal wall prolapse, posterior vaginal wall prolapse, prolapse of the apical segment of the vagina or their combination.
- Studied involving a subset of patients at stage 0 defined by Pelvic Organ Prolapse Quantification (POP-Q) will be excluded;
- Studies involving only a subset of POP participants will be excluded.

### 2.2 Types of interventions

Intervention group of the eligible trial must include 1 conservative treatment or their combinations to treat POP, which may include:

- Lifestyle interventions, like weight loss, avoiding heavy lifting, and avoiding coughing, etc.
- Devices, like support pessaries and space filling pessaries, etc.
- Physical therapies, like physical activity, cognitive behavioral therapy, pelvic floor muscle training, bladder training, bowel habit training, coordination training, biofeedback, electrical muscle stimulation, etc.

Control group can be inactive intervention, such as standard care or a waiting list, or an active intervention, such as a different kind of conservative treatment or a combination of several different conservative treatment.

RCTs with surgery or pharmacological treatment existed in any arm will be excluded. RCTs comparing an intervention with a different variant of the same intervention will be excluded.

#### 2.2.1 Types of outcome measures

##### 2.2.1.1 Primary outcomes

Specific POP-related symptom (e.g. Pelvic Organ Prolapse Symptom Score)

##### 2.2.1.2 Secondary outcomes

- Severity of prolapse (e.g. anatomical changes measured by POP-Q or pelvic floor functional changes measured by ultrasound),
- Prolapse-related and general health-related quality of life,
- Other non-POP-specific symptoms (e.g. urinary symptoms, bowel symptoms, or sexual symptoms).

### 2.3 Search methods for identification of studies

We will conduct electronic searches in 3 databases from their inception date to July 2019, including PubMed, EMBASE, and the Cochrane Library.

Search strategy will be drawn up in accordance with the Cochrane handbook guideline. Research strategy of PubMed is presented in Table 1, and will be modified to search in the other databases. Only studies reported in English will be included.

**Table 1:**
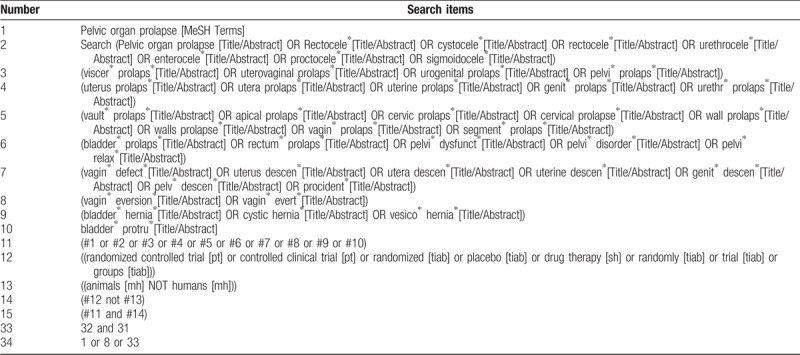
Search strategy used in the PubMed database.

### 2.4 Data collection and analysis

#### 2.4.1 Selection of studies

**Figure 1:**
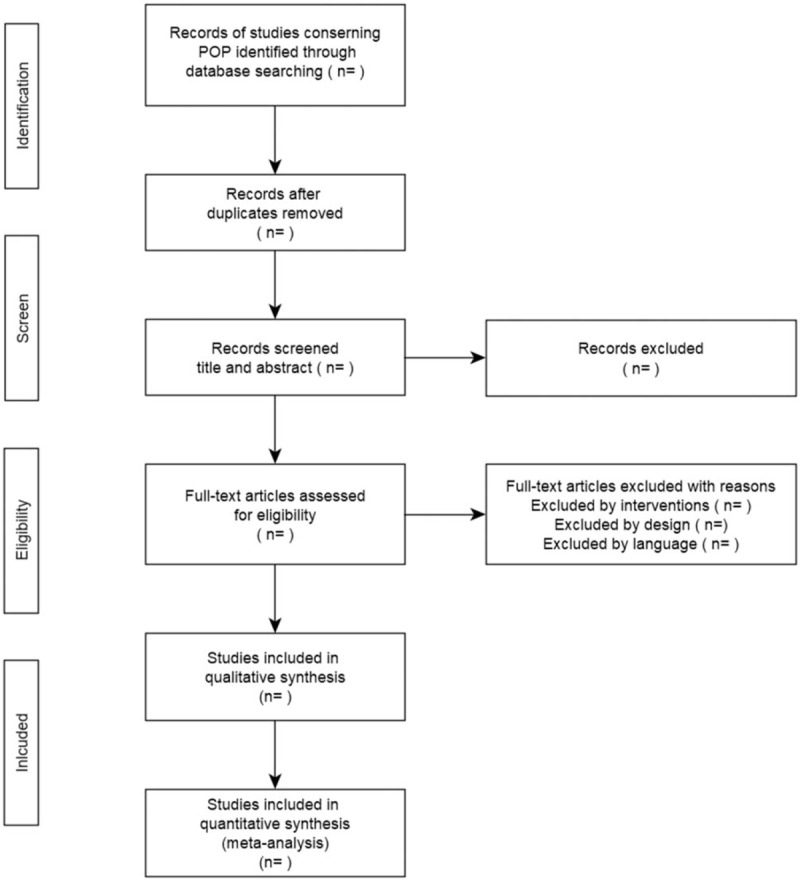
Study flow diagram.

The articles searched from databases will be imported into EndNote X9 software, with the help of which duplicated ones are removed. Two authors (YS and HC) will screen the titles and abstracts independently to select potentially eligible articles and then go through the full text of those articles to exclude ineligible ones according to predetermined criteria. During the process, any disagreement between the 2 authors will be discussed with ZL to make a final decision. The screening process is presented in Figure 1 as required by PRISMA.

#### 2.4.2 Data extraction and management

Two authors (RJ and JY) will extract the data separately, and a data extraction form will be applied in collection. Before actual extraction, we will put the data of 2 or 3 articles into the form to make sure the absence of obvious distinctions.

The data will be extracted included: administration details of the article, methods of the trial, participants enrolled, interventions applied, controls used, primary and secondary outcomes, measurements, and results, etc. Any disagreement during the process will be discussed with ZL.

#### 2.4.3 Assessment of risk of bias in included studies

The risk of bias will be assessed by 2 independent authors (YS and XZ) in accordance with Cochrane Collaboration Risk of Bias Tool in the domains of random sequence generation, allocation concealment, blinding of participants and personnel, blinding of outcome assessment, incomplete outcome data, selective reporting, and other bias. Each trial will be evaluated as low risk, high risk, and unclear risk, based on the conduct process of the trials. Any disagreement during the process will be discussed with ZL.

#### 2.4.4 Unit of analysis issues

If results of the same trial were multiply published in several journals, the article will be excluded. If different outcomes of the same trial were published in different journals, the data will be extracted and integrated into one.

#### 2.4.5 Dealing with missing data

For trials with incomplete or unclear data reported, the correspondent author of the article will be contact by phone or E-mail to seek for possible help. An intention-to-treat analysis will be performed when possible and a sensitive analysis will be made to confirm the consistence.

#### 2.4.6 Assessment of heterogeneity

Before synthesizing the data, the heterogeneity of the studies will be tested by *I*^2^ statistic. The studies with an *I*^2^ value less than 50% will not be regarded as of heterogeneity and the data extracted will be synthesized; while those with an *I*^2^ value more than 50% will be considered as of heterogeneity, the reasons of which might be sought by subgroup analysis or from clinical and design aspects.

#### 2.4.7 Assessment of reporting biases

Funnel plots will be made to detect whether there is report bias if over 10 studies are meta-analyzed under 1 outcome.

#### 2.4.8 Data synthesis

The data will be synthesized in RevMan V.5.3 if possible. MD or SMD will be used to analysis continuous data, while RR, dichotomous data. Fixed-effects model will be applied if *I*^2^ value is less than 50%, and random-effects model, if *I*^2^ value is between 50% and 75%. When *I*^2^ value exceeds 75%, a descriptive analysis rather than a meta-analysis will be made, and we will try to explore the reasons behind by subgroup analysis, or from clinical and design aspects.

#### 2.4.9 Measures of treatment effect

RevMan V.5.3 will be used to analyze the data extracted. Mean difference (MD) or standard MD (SMD) with 95% CI will be used to analysis continuous data, while risk ratio (RR) with 95% CI will be used to analyze dichotomous data.

#### 2.4.10 Subgroup analysis

Since conservative treatments are consisted of several different interventions, we plan to make subgroup analysis according to different therapies. Subgroup analysis might also need to be made to seek the cause of heterogeneity, if necessary.

#### 2.4.11 Sensitivity analysis

We will perform sensitivity analysis to test the robustness of review process if needed. Studies with high or unclear risk of bias in allocation concealment domain will be included and excluded studies with if needed, and the results will be discussed accordingly.

#### 2.4.12 Grading the quality of evidence

Quality of the evidence will be judged according to Grading of Recommendations Assessment, Development and Evaluation working group methodology in the domains of risk of bias, consistency, directness, precision, publication bias, and additional points, based on which the assessments will be classified as high, moderate, low, or very low.

## 3 Discussion

Though POP rarely cause mortality in clinic, the symptoms of urogenital and gastrointestinal systems can be rather bothersome to female patients and greatly reduce their quality of life.^[15]^ The prolapse of vaginal segments is related to different pelvic organs, like bladder, rectum, or uterus, etc. Previous research indicated that anterior vaginal wall prolapse occurred the most frequently in clinic, followed by posterior vaginal wall and apical prolapse,^[7,16]^ usually in combination.^[17]^ Besides, the cost of POP put large burden to the healthcare system.^[18]^ Several systematic reviews published before to evaluate conservative treatments for POP years ago, and results indicated that the effectiveness and safety of them needed to be supported by more trials.

Since conservative treatment is defined as non-pharmacological and non-surgical treatment, and consist of a variety of therapies, the search strategy will be focus different names and forms of the disease, rather than the intervention itself. Ineligible trials will be excluded after research by hands.

There exists limitation in this systematic review in that some articles may be missed because only articles in English will be enrolled. Since conservative treatments included a variety of specific therapies, it will be a big challenge as how to categorize them appropriately, synthesize the data and report the results.

## Acknowledgments

Great appreciation and respects to Simi Shao worked in National Library of China, for her patience and guidance in the development of search strategies.

## Author contributions

ZL and YS conceived the study. YS, ZL, HC and YL developed the search strategies. RJ, JY and XZ identified the key words and screened them in the three databases to simplify the search strategies. YS wrote the first draft and revised by ZL, HC and YL. All authors approved the publication of the protocol. The funder has no role in the design and conduction of the systematic review.

Zhishun Liu orcid: 0000-0001-7570-8917.
